# Histological findings of an autologous dermal fat graft implanted onto the pectoralis major muscle of a rat model

**DOI:** 10.1007/s12282-014-0523-5

**Published:** 2014-02-27

**Authors:** Tadao Mizoguchi, Yuko Kijima, Munetsugu Hirata, Koichi Kaneko, Hideo Arima, Akihiro Nakajo, Michiyo Higashi, Kazuhiro Tabata, Chihaya Koriyama, Takaaki Arigami, Yoshikazu Uenosono, Hiroshi Okumura, Kosei Maemura, Sumiya Ishigami, Heiji Yoshinaka, Yoshiaki Shinden, Shinichi Ueno, Shoji Natsugoe

**Affiliations:** 1 Department of Digestive Surgery, Breast and Thyroid Surgery, Graduate School of Medical and Dental Sciences, Kagoshima University, 8-35-1, Sakuragaoka, Kagoshima, 890-8520 Japan; 2 Division of Human Pathology, Department of Oncology, Graduate School of Medical and Dental Sciences, Kagoshima University, Kagoshima, Japan; 3 Department of Epidemiology and Preventive Medicine, Graduate School of Medical and Dental Sciences, Kagoshima University, Kagoshima, Japan

**Keywords:** Breast cancer, Breast-conserving surgery, Oncoplastic surgery, Free dermal fat graft, Rat model, Cosmesis, VEGF, Apoptosis

## Abstract

**Background:**

The aim of this study was to investigate the maintenance of volume as a spacer by comparing vascular supply and apoptosis in an implanted autologous-free dermal fat graft (FDFG) and free fat graft (FFG). An autologous FDFG is a material used in plastic surgery and oncoplastic breast surgery that is ideal for immediate volume replacement after partial mastectomy because of its easy availability and minimal invasion of the donor site; however, immunohistochemical findings and survival procedures have not yet been reported.

**Methods:**

An experimental protocol using a unique animal model was designed for the present study. The expression of vascular endothelial growth factor (VEGF) was measured in FDFGs and FFGs implanted onto the pectoral major muscle of Wistar rats. Thirty Wistar rats were divided into two groups and postoperatively 1, 2, 4, 8, and 16 weeks (POW1, 2, 4, 8, 16). Six samples from three rats in each group were used as control samples (POW0).

**Results:**

The thickness of the implanted FDFG was not significantly different from the control sample at POW1, 2, 4, 8, and 16 between FDFG and FFG group; however, the thickness at POW8 and 16 was significantly lesser in the FFG group than in the control samples.

The average proportion of fatty tissue to whole tissue ranged from 34.2 to 48.6 % in the FDFG group and from 57.2 to 76.7 % in the FFG group during the observation period; however, there was no significant difference in the proportion of fatty tissue between these two groups. There were no significant differences between the average number of VEGF-positive cells in the FDFG group and the FFG group at POW1, 2, 4, 8, and 16. The average number of TUNEL-positive cells in the early period at POW1 was significantly lower in the FDFG group than in the FFG group.

**Conclusions:**

This rat model was useful for investigating the mechanisms of angiogenesis, apoptosis, structure maintenance, and fibromatous changes. From the present experimental study, we believe that FDFG is one of the most convenient materials currently available to repair small defects at the time of BCS even in the clinical field.

## Introduction

Oncoplastic breast surgery, in combination with oncological and plastic procedures during surgical treatment for early breast cancer, has become popular, especially in Western countries [1–3]. There are currently two fundamentally different approaches: volume replacement procedures that combine resection with immediate reconstruction of the defect and volume-reduction procedures that combine resection with a variety of different breast reduction and reshaping techniques. We previously reported several clinical results of oncoplastic surgery combining partial mastectomy and immediate volume replacement using an autologous free dermal fat graft (FDFG) in Japanese patients with early breast cancer on the upper-inner or central area of small breasts [4]. Such a technique achieved better cosmetic results than the transposition of residual breast tissue, and more convenient results than using mini-flap of the latissimus dorsi [5, 6]. However, few studies have referred to an experimental model of FDFG, and FDFG implanted onto the pectoralis major muscle according to the clinical procedure at the time of breast-conserving surgery (BCS) has not yet been evaluated histologically.

Although several studies have reported transplantation using free fat grafts (FFGs) or fat injections, we could not find any literature regarding an investigation of FDFG. The purpose of this study was to histologically evaluate FDFG implanted onto the major pectoral muscle to evaluate the condition of fatty cells in animal models, and to predict the role of oncoplastic surgery using FDFG at the time of BCS.

## Methods

### Animals

Thirty-six 7-week-old male Wister rats weighting 200 g were bred in wire cages until their weight increased to 600 g (640–810 g). Thereafter, we could easily perform autologous implantation of FDFG and a FFG.

### Harvest and transplantation of FDFGs and FFGs

**Fig. 1:**
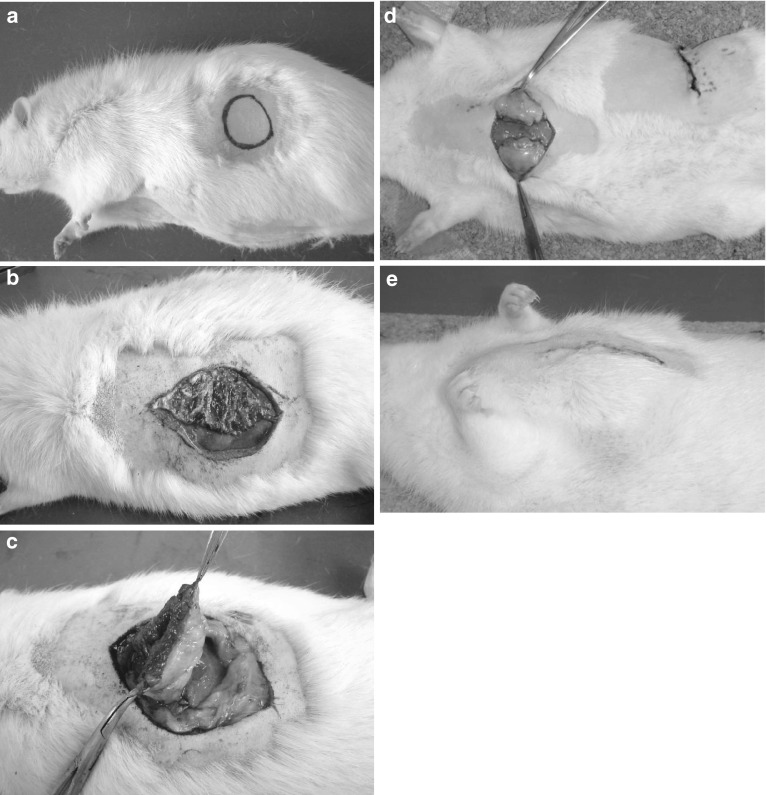
Implantation of FDFG onto the major pectoral muscle. **a** Incision areas were marked on the lateral abdomen. **b** In situ de-epithelialization, followed by sharp dissection and trimming to a thickness of 10 mm, was performed using a knife. **c** An FDFG with ellipse-shaped dermis and 10-mm thickness of subcutaneous fatty tissue were harvested. Then, it was divided into two pieces as columns with a base of 2 cm in diameter. **d** Two FDFGs were implanted onto the surface of the pectoralis major muscle and fixed peripherally using absorbable sutures to connect the dermis with the surface of the major pectoral muscle. **e** At the end of the operation procedure

After 20-week-old rats were anesthetized by inhaling diethyl ether, incision areas were marked on the lateral abdomen (Fig. 1). To harvest FDFGs, in situ de-epithelialization, followed by dissection and trimming to a thickness of 10 mm, was performed using a knife (Fig. 1b, c). FDFGs were divided into two pieces as columns, with a base 2 cm in diameter, which were then bilaterally implanted onto the surface of the pectoral major muscle in the same manner as clinical BCS. FDFGs were fixed peripherally using absorbable sutures to connect the dermis with the surface of the major pectoral muscle (Fig. 1d, e). FFGs were harvested from the lateral abdomen. Two blocks of columns, with a base 2 cm in diameter, were implanted onto the surface of the major pectoral muscle. On the edge of FFG, some sutures were added for fixation in the same manner as the FDFG group.

### Sample collection

Three rats implanted with FDFGs and FFGs were euthanized 1, 2, 4, 8, and 16 weeks postoperatively, respectively (providing samples for POW1, POW2, POW4, POW8, and POW16). The implanted FDFG/FFGs with the pectoralis major muscle and skin were resected in a lump as one block. Six samples, two samples from one rat, were collected for each postoperative period in the FDFG and FFG groups. After being fixed immediately in neutral buffer formalin for 48 h, they were embedded in paraffin. Samples were cut at a thickness of 3 μm and were fixed onto glass slides retreated with 3-aminopropyltriethoxysilane (Matsunami Glass Ind., Ltd. Japan). Six FDFG samples and six FFG samples were collected as control samples, respectively. There were immediately fixed in neutral buffer formalin for histological evaluation.

### Histological examination

A total of 6 samples in the FDFG and FFG groups 1, 2, 4, 8, 16 weeks postoperatively were analyzed using hematoxylin and eosin (HE) and immunohistochemical staining. Six samples obtained at POW0 from the two groups were used as control samples. One representative section in the largest portion of each FDFG or FFG sample was selected for histological examination.

At 40× objective on HE staining, we microscopically measured the thickness of FDFG or FFG as a whole. At 100× objective, 5 fields were randomly selected from HE-stained sections, and fatty tissue areas were measured using ImageJ (Wayne Rasband, National Institues of Health, Bethesda) [7]. The proportion of fatty tissue to whole tissue was recorded in each sample after measuring fatty tissue or fibrous tissue.

To evaluate microvascular angiogenesis and the apoptosis of adipose cells on each dimension of FDFG/FFG, we selected 5 fields including a central area, a pectoralis major muscle side, a skin side, and the lateral sides of FDFG/FFG for immunohistochemical staining. Representative sections were stained using a streptavidin–biotin–peroxidase supersensitive kit (Dakopatts, Glostrup, Denmark), with a rat monoclonal anti-VEGF antibody (Santa Cruz Biotechnology Inc., America) for angiogenesis at a concentration of 1:100. The endogenous peroxidase activity of specimens was blocked by immersing the slides in a 3 % hydrogen peroxidase-methanol solution for 30 min at room temperature. After washing three times with PBS for 5 min each, sections were treated with 1 % bovine serum albumin for 30 min to block non-specific reactions at room temperature. Then, sections were incubated with the primary antibody to VEGF (1:100) at 4 °C overnight and were subsequently stained with secondary antibodies for 30 min. Sections were incubated with the avidin–biotin complex for 60 min and reactions were visualized using diaminobenzidine tetrahydrochloride for 2 min. Sections were rinsed briefly in water and counterstained with hematoxylin for 30 s. An area was selected randomly to evaluate the expression of VEGF on each section and measure the contribution of VEGF-positive cells by ImageJ (magnification, 100×). TUNEL staining (TaKaRa Bio, In Situ Apoptosis Detection Kit) was used to detect apoptotic cells [8–10]. Positive areas were randomly selected to count the number of TUNEL-positive adipose cells (magnification, 400×).

### Statistical analysis

We compared the thickness of each postoperative period, and the expression of VEGF-positive cells and TUNEL-positive cells between the two groups using a two-sided Student’s *t* test. A *p* value of <0.05 was considered significant. Data were expressed as the mean ± SD.

## Results

**Fig. 2:**
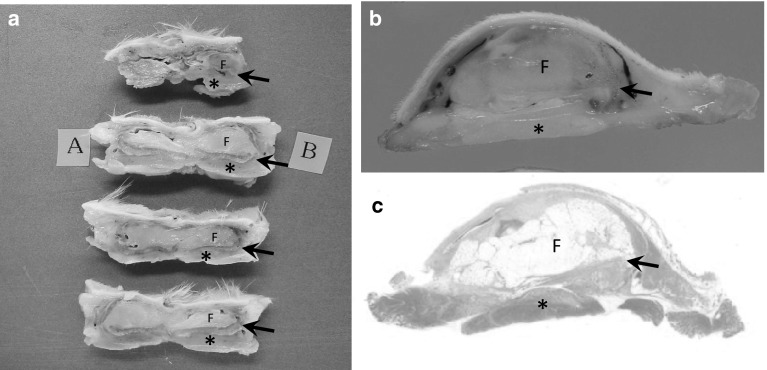
Macroscopic and microscopic findings of the resected sample (FDFG group, POW1) resected with anterior chest tissue (skin, subcutaneous tissue, pectoral muscle and ribs) and the implanted FDFG as one sample. **a** A sample after 48-h fixation of neutral buffer formalin was cut into serial sections. You can see a maximum sectioned surface of the FDFG (sections A and B). *Arrow* pointed the dermis attached to the fatty tissue. **b** A section with maximum surface of the a FDFG. Such two samples were collected from one rat. **c** A total of 6 samples in the FDFG and FFG groups 1, 2, 4, 8, 16 weeks postoperatively were analyzed using hematoxylin and eosin (HE) and immunohistochemical staining. (×4, HE staining). *F* FDFG, *asterisk* pectoral muscle

**Fig. 3:**
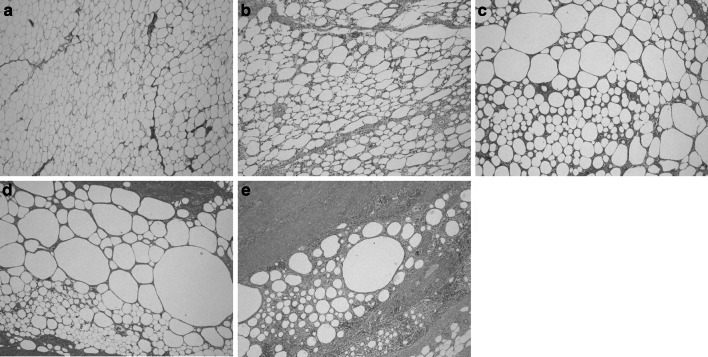
Histological findings of the implanted FDFG, HE staining (×40). **a** POW1, **b** POW2, **c** POW4, **d** POW8, **e** POW16

A total of 72 samples from 36 rats were collected and evaluated as follows: 18 rats were POW0, 1, 2, 4, 8, and 16 in the FDFG group, 18 were POW0, 1, 2, 4, 8, and 16 in the FFG group. No necrotic changes or infections were observed. On HE staining, fatty tissue and eosinophilic fibrous tissue were recognized separately (Fig. 2). Fatty tissue was recognizable at the end of the observation period and infiltrating lymphocytes, macrophages, and fibrosis appeared from POW1 to POW16 (Fig. 3).

### Thickness of implanted samples

**Fig. 4:**
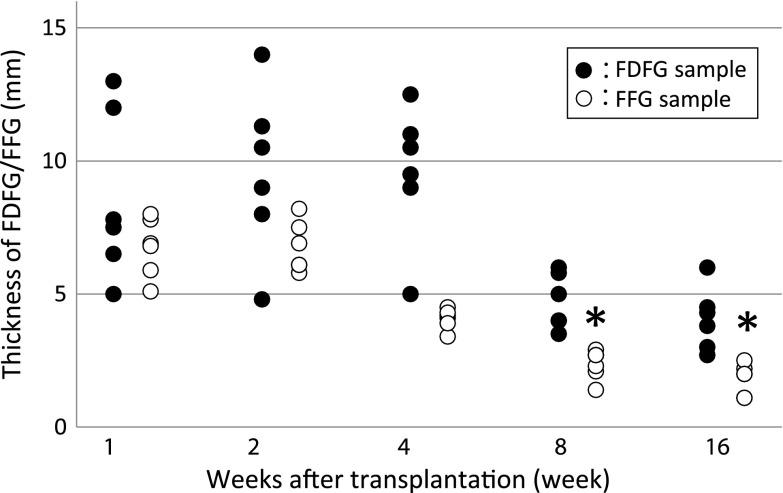
Thickness of the implanted FDFG/FFG after implantation. *Asterisk* indicates that the average thickness of the implanted sample was significantly lesser than that of the control sample (POW0)

The average thickness of the implanted FDFG was 8.6 mm (6.5–13.0), 9.8 mm (4.8–14.0), 9.3 mm (5.0–12.5), 4.5 mm (3.5–6.0), and 3.7 mm (1.5–6.5) in the FDFG group at POW1, 2, 4, 8, 12, and 16, respectively. The average thickness was 6.8 mm (5.1–8.0), 6.8 mm (5.8–8.2), 4.1 mm (3.4–4.5), 2.4 mm (1.4–2.9), 1.8 mm (1.1–2.5) in the FFG group at POW1, 2, 4, 8, and 16, respectively (Fig. 4). In the FFG group, the thickness of the POW8 and POW16 samples was significantly lesser than that of the control sample (10 mm). On the other hand, the thickness of the implanted FDFG at any postoperative period was not significantly different from that of the control sample (Fig. 4). At each period, there was no significant difference in thickness between the FDFG and FFG groups.

### Proportion of fatty tissue

**Fig. 5:**
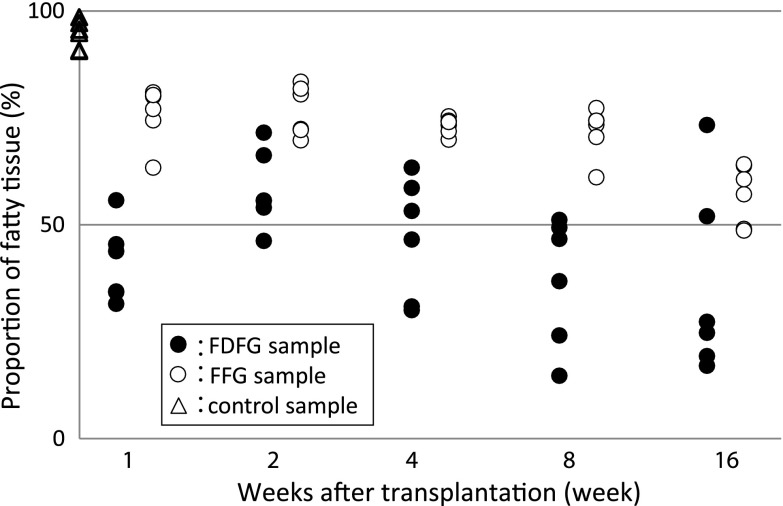
Proportion of fatty tissue of the implanted FDFG/FFG after implantation. There were no significant differences between each period and control samples, or between FDFG and FFG samples at each period

The average proportion of fatty tissue to whole tissue was 40.8 % (31.5–55.7), 51.2 % (10.4–71.5), 48.6 % (30.0–63.3), 45.0 % (14.7–69.4), and 34.2 % (4.2–74.3) in the FDFG group, and 76.0 % (63.3–80.9), 76.7 % (69.7–83.4), 73.1 % (69.9–75.3), 71.8 % (61.1–77.3), and 57.2 % (48.6–64.1) in the FFG group at POW1, 2, 4, 8, and 16, respectively. Also, there was no significant difference between each postoperative value and the control value in both groups. At each period, there was no significant difference in the proportion of fatty tissue between the FDFG and FFG groups (Fig. 5).

### Immunohistochemical examination

**Fig. 6:**
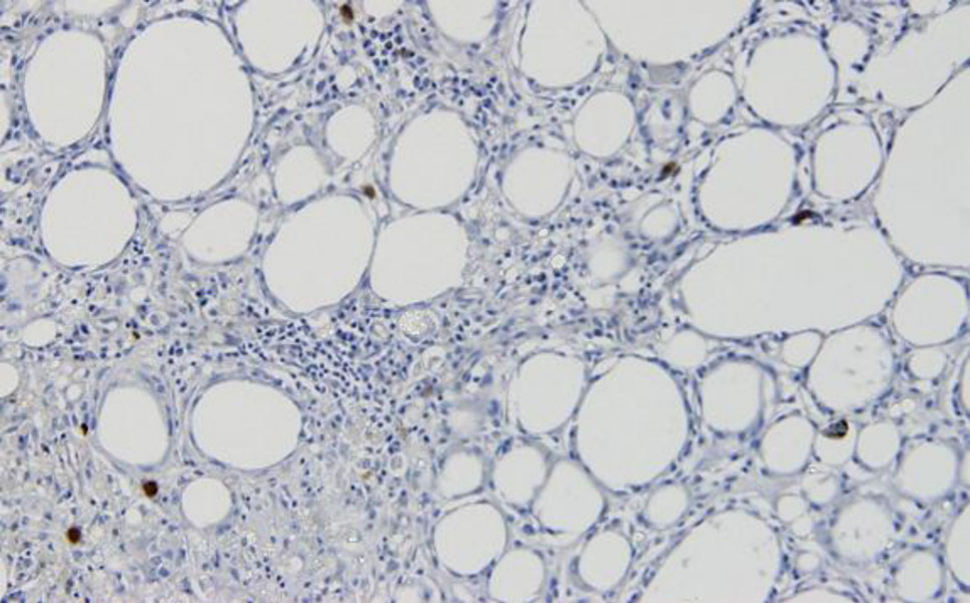
Expression of VEGF-positive cells immunohistochemical staining for VEGF. VEGF-positive cells were found in the graft area (in the POW4 sample from the FDFG group, ×200)

VEGF-positive cells were observed in all sections. They could be seen in the extracellular matrix around adipose cells (Fig. 6). The average number of VEGF-positive cells in the central area of the grafts was measured by the ImageJ System. In control samples, the average number was 536.1 pixels (262.0–1318.7). When samples were compared between the FDFG and control groups, the average number in the FDFG group was 25019.5 pixels (6686.7–74445.0) (*p* = 0.0368), 45258.8 pixels (18822.0–62806.0) (*p* < 0.0001), 32784.2 pixels (18194.3–48038.7) (*p* < 0.0001), 34009.5 pixels (24508.7–53673.3) (*p* < 0.0001), and 32821.9 pixels (8779.7–54988.3) (*p* = 0.0019) at POW1, 2, 4, 8, and 16, respectively.

**Fig. 7:**
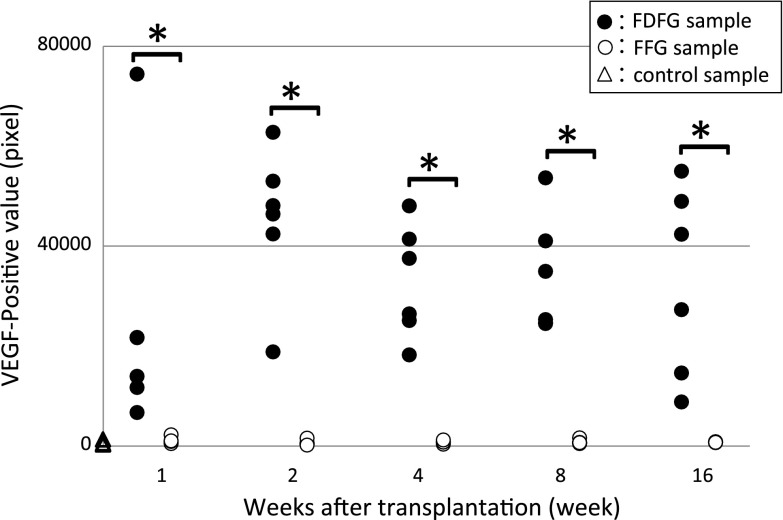
Expression of VEGF-positive cells in the central area of the graft area. *Asterisk* indicates that the average VEGF-positive value was significantly higher in the FDFG group than in the FFG group at each postoperative period

The average number of VEGF-positive cells in the FFG group was 1055.3 pixels (475.7–2228.0) (*p* = 0.1120), 601.8 pixels (171.0–1528.7) (*p* = 0.8027), 770.9 pixels (651.3–1194.7) (*p* = 0.2656), 786.3 pixels (441.7–1615.3) (*p* = 0.3140), and 752.3 pixels (622.0–857.3) (*p* = 0.2212) at POW1, 2, 4, 8, and 16, respectively. VEGF-positive cells were higher in the FDFG group than in the control group at all periods. The average number of VEGF-positive cells was significantly higher in the FDFG group than in the FFG group at POW1, 2, 4, 8, and 16 (Fig. 7). The same results were seen for the other four areas: the pectoralis major muscle side, the skin side, and the horizontal sides of FDFG/FFG (data not shown).

**Fig. 8:**
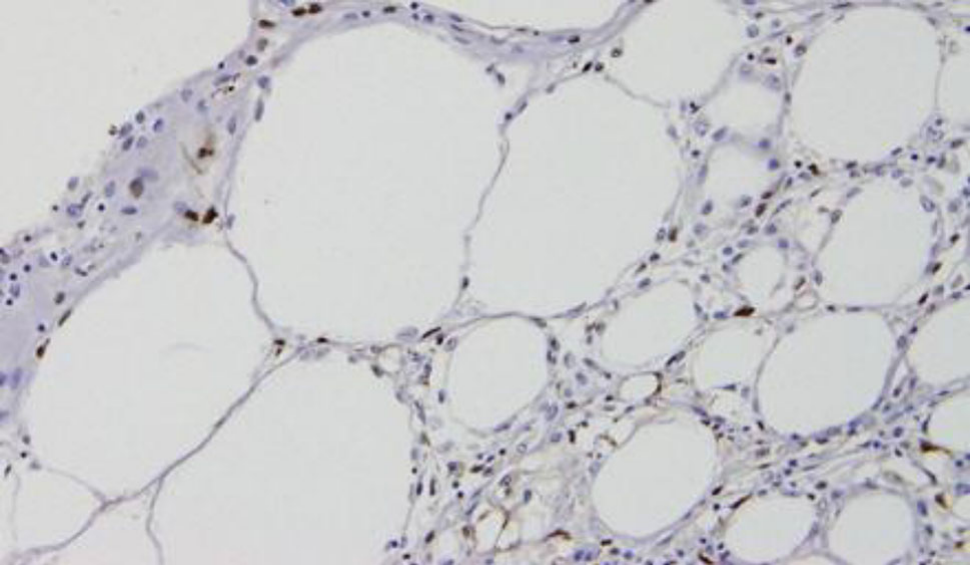
Expression of TUNEL-positive cells. TUNEL-positive cells were found in the graft area (in the POW4 sample from the FFG group, ×200)

**Fig. 9:**
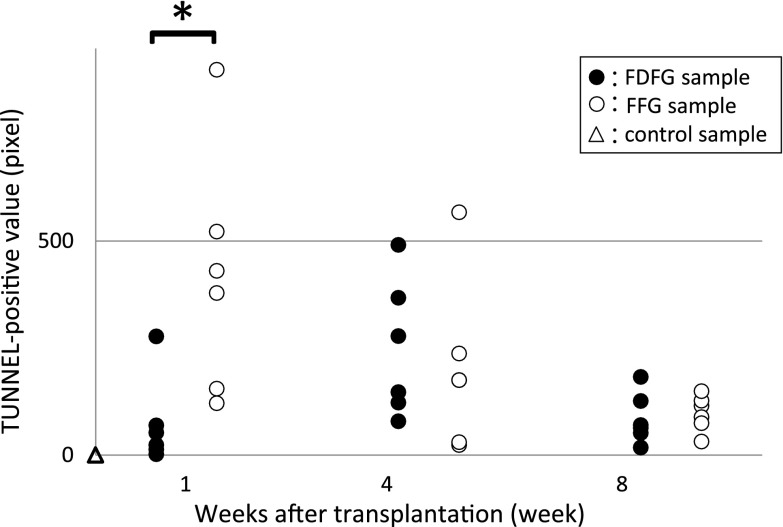
Expression of TUNEL-positive cells in the central area of the graft area. *Asterisk* indicates that the average TUNEL-positive value was significantly higher in the FFG group than in the FDFG group

TUNEL-positive cells were observed in all sections of the FDFG and FFG groups, but not in the control group. TUNEL-positive cells were sporadically found in fatty tissue (Fig. 8). The average value of TUNEL-positive cells using the ImageJ system in the FDFG group was 72.7 pixels (2.0–277), 247.3 pixels (79–491), and 84.8 pixels (17–182) at POW1, 4, and 8, respectively, while in the FFG group, this value was 417.7 pixels (121–900), 201.2 pixels (23–567), and 97.3 pixels (31–149) at POW1, 4, and 8, respectively. The average number of TUNEL-positive cells in the early period at POW1 was significantly lower in the FDFG group than in the FFG group (Fig. 9).

## Discussion

FFGs have been used for soft tissue augmentation, because they show many qualities of an ideal filler. They are autologous and completely biocompatible [11–13]. A free autologous fat graft has been suggested for augmentation in the treatment of unilateral vocal cord palsy and for the cosmetic improvement of facial contours [8, 10, 14–16]. The survival of fat grafts depends on many factors, especially revascularization [17]. Baran et al. [18] showed that the resorption of a fat graft in a rabbit model was reduced in areas where vascularity was supplied. In another study, the application of autologous platelet-rich plasma was shown to enhance FFG survival in rabbits [12].

Autologous FDFG has been sporadically used for soft tissue augmentation. In particular, autologous FDFG has been suggested to be an ideal reconstructive technique in the field of surgical treatment for head and neck disease, because it is easy, inexpensive, and simple [19–21]. Small-volume FFGs have been reported to survive by osmotically obtaining a nutrient supply until a vascular supply can be established, such that the structure of transplanted tissue varies in success. Lexer reported that healing occurred with a gradual disintegration of the transplanted tissue and coincident regeneration of the homologous tissues of the recipient from those in the immediate vicinity [22]. FDFG was grafted onto the dorsal surface of the ears in a swine model. Although fat disappeared slightly with a significant part of it being replaced by fibrous tissue, FDFG implanted in the ears of growing pigs not only survived, but also grew [23]. Another researcher reported the same results using a pig model, in which FDFG harvested from the back was implanted into the rectus muscle [24]. Such experimental findings suggest that FDFG adipose tissue is at least partially replaced by host fibrous tissue, but that the texture and pliability of the graft are also clinically preserved for years after implantation.

We introduced an immediate volume replacement technique using FDFG because of its safety and convenience for BCS, which resulted in excellent cosmetic results without complications [4–6]. We found that it was effective in patients with small breasts and a slim body; the smooth dermal surface was placed over the pectoralis major muscle. There have been only a few reports on the clinical characteristics of FDFG implanted into partial defects at the time of BCS and on the pathologic findings of implanted FDFG [25]. We previously reported an interesting finding from a pathologic evaluation of core needle samples from implanted FDFG after BCS in a clinical series. Fatty tissue was observed at a proportion of over 50 percent in CNB samples from patients at over 48 months postoperatively, which was a greater value than we expected at the time of BCS [26]. These findings are different from those of other researchers who observed absorption and fibrous degeneration. From this clinical experience and pathological studies, we hypothesized that BCS and immediate volume replacement using FDFG may have established some ideal environment for implanted FDFG being surrounded breast or fatty tissue on the surface of the major pectoral muscle. However, there was a limit to clear when vascularization and/or necrosis in the implanted FDFG occurred. What is an important method for adding a dermis to a fatty graft? It is very difficult for us to obtain several samples from one patient at different postoperative periods. An animal model in which we can easily repeat a condition such as immediate volume replacement of a partial defect using autologous FDFG has not yet been established.

In this study, we developed a rat model implanted with FDFGs and FFGs. We showed that the histological findings of implanted FDFGs on the pectoralis major muscle resembled the clinical results we previously reported on FDFGs implanted onto partial breast defects with early breast cancer. These findings were not found in other studies. We also hypothesized from clinical experience that implanted FDFGs may undergo mild resorption and degeneration to fibrous tissue, but that the volume they filled may be maintained long after surgery as a “spacer”. In the present study, the thickness of the implanted graft gradually decreased, but was less affected in the FDFG group than in the FFG group (*p* = 0.0166). The proportion of fatty tissue was slightly lower in the FDFG group than in the FFG group although it was not significant. From pathological findings in the present study, the larger thickness in size of FDFG group depends on the volume of parenchymal tissue, such as fibrosis, infiltration or of lymphocyte except fatty tissue. Attaching dermis to fatty tissue may influence something to the parenchymal tissue resulting in keeping better volume than that without any dermis. If the greater volume of FDFG group depends on the volume of parenchymal tissue, the results of TUNNEL method might show the positive relationship between apoptotic cells and parenchymal volume of implanted FDFG/FFG. The fact that thickness of the grafts was measured in histologic sections implies that the shrinkage and other eventual changes which occur with formalin fixation and the various tissues dehydration steps would have made these results inaccurate from “real state”. We did not measure the thickness at the time of explanation. To avoid producing gaps between the grafts and surrounding tissue, such as pectoral muscle, fatty tissue, and skin, we did not make the direct intervention before an adequate fixation. However, we should have been able to make another experimental rat model for the right measurement of the fresh sample but not for histological examinations. The following examination may be needed.

Higher expression of VEGF and lower apoptosis was measured in the FDFG group than in the FFG group. These results suggest that FDFG may maintain volume at least until the end of the observation. Although the proportion of fatty tissue was slightly lower in the FDFG group than in the FFG group (without a significant difference), the total volume of implanted tissue was larger in the FDFG group. Various factors for angiogenesis, such as the VEGF family, have been reported to play an important role in the revascularization of the graft [27]. In the local wound environments, endogeneous cells play the multiple roles in producing VEGF. Platelets arrive first on Day “0” of wounding, followed by a peak of macrophages at Day 2. Endothelial cells begin to migrate at Day 2 and new capillary endothelium can be seen between Days 3 and 4. By Day 5, new collagen is produced from the fibroblasts. The initial cells that release VEGF are platelets which enter the wound after debridement. In addition, macrophages release VEGF which stimulates endothelial cells to proliferate and migrate. VEGF has been shown to stimulate keratinocyte migration and collagen production via fibroblasts. VEGF secretion also induces release of other growth factors which further stimulate healing [28]. They also reduce the rate of apoptosis in adipose cells, improve fat cells, and determine how fat grafts obtain their vascular supply and why they keep decreasing in volume and weight during long-term follow-ups. In the results of their study, dead fat cells and fat drops were removed and weight loss occurred 30 days after the transplantation. Revascularization and the suppression of apoptosis of the implanted FDFG may be related with the longer maintenance of their volume. Also, the application of VEGF may improve graft viability and the ability to maintain volume as a spacer. Chandarana et al. [29] reported that autologous platelet adhesives improved grafts in patients who received superficial parotidectomy and immediate reconstruction using FDFG. We should add the comparisons in the expression of factor 8 and micro vessel density using this unique animal model in the near future.

Our new rat model in which FDFG was implanted onto the pectoralis major muscle was very useful for investigating the mechanisms of angiogenesis, apoptosis, structure maintenance, and fibromatous changes. From the present experimental study, we believe that FDFG is one of the most convenient materials to repair small defects at the time of BCS.

## Conclusion

We histologically investigated the role of attaching a dermis to FFGs in an autologous transplantation from the lateral abdomen onto the major pectoral muscle in a rat model. FDFG maintained blood flow and apoptotic resistance.
